# Effects of Biofilm Formation on Gastrointestinal Tolerance, Mucoadhesion and Transcriptomic Responses of Probiotics

**DOI:** 10.1002/fsn3.70206

**Published:** 2025-05-14

**Authors:** Wang Gao, Huijuan Jing, Bo Qiu, Shuobo Zhang, Jingyi Zhang, Lvwan Xu, Furong Ba, Siyuan Xie, Xiao‐Man Liu, Lanjuan Li, Mingfei Yao

**Affiliations:** ^1^ Jinan Microecological Biomedicine Shandong Laboratory Jinan China; ^2^ State Key Laboratory for Diagnosis and Treatment of Infectious Diseases, National Clinical Research Center for Infectious Diseases, Collaborative Innovation Center for Diagnosis and Treatment of Infectious Diseases, The First Affiliated Hospital, School of Medicine Zhejiang University Hangzhou China; ^3^ Shandong First Medical University & Shandong Academy of Medical Sciences Jinan China; ^4^ Central Laboratory Shandong Provincial Hospital Affiliated to Shandong First Medical University Jinan Shandong People's Republic of China

**Keywords:** biofilm, gastrointestinal tolerance, intestinal adhesion, probiotics

## Abstract

Probiotic health benefits may be affected by decreased viability during food storage and gastrointestinal transit. Although microencapsulation is an effective protective strategy, its application to probiotics is limited. Currently, research on probiotic biofilms is expanding, with these biofilms being recognized as the fourth generation of probiotics. This study aimed to investigate the effects of biofilm formation on gastrointestinal tolerance and mucoadhesion of three different probiotics: *Ligilactobacillus salivarius* Li01 ( *L. salivarius* Li01), *Bifidobacterium longum* ( *B. longum* ), and *Bifidobacterium pseudocatenulatum* ( *B. pseudocatenulatum* ). Biofilm growth was markedly inhibited by low pH and high bile salt concentrations. The formation of biofilms greatly improved the survival of all three strains under simulated gastrointestinal conditions. The biofilms increased intestinal adhesion and surface hydrophobicity in *B. longum* and *L. salivarius* Li01, while reducing adhesion in *B. pseudocatenulatum* due to decreased hydrophobicity. Moreover, transcriptomic analysis of *L. salivarius* Li01 identified 157 differentially expressed genes, enriched in pathways including ABC transporters, quorum sensing, purine metabolism, arginine biosynthesis, the phosphotransferase system (PTS), RNA polymerase, and the NOD‐like receptor signaling pathway. In conclusion, the formation of biofilms enhances gastrointestinal tolerance and intestinal adhesion of probiotics, presenting great applied potential in increasing the efficacy of probiotics.

## Graphical abstract

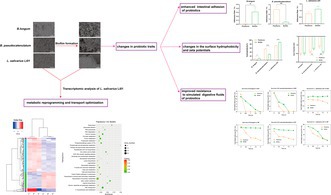

## 1 Introduction

Probiotics refer to live microorganisms that, when consumed in sufficient quantities, provide health advantages to the host (Hill et al. 2014). Probiotics have been intensely studied due to their benefits to host health since their discovery (Barzegar et al. 2023; Lai et al. 2023; Suez et al. 2019). In recent years, probiotic biofilms have gained increasing attention, and they are considered as the most advanced fourth‐generation probiotics (Deng et al. 2020; Yang et al. 2024). As we all know, bacteria primarily exist in two states: planktonic and biofilm states (Li et al. 2023). The planktonic state describes free‐floating bacteria in liquids, typically characterized by rapid multiplication and dispersal in resource‐rich environments (Rumbaugh and Sauer 2020). Biofilms are structured microbial communities that adhere to surfaces, with cells encased in a self‐produced extracellular polymeric substance (EPS) matrix, which constitutes the dominant form of bacterial existence in nature (Gao et al. 2022). The EPS matrix consists of exopolysaccharides, extracellular nucleic acids, proteins, lipids, and other biomolecules (Karygianni et al. 2020). Most studies focus on biofilms formed by pathogenic bacteria, as they exhibit greater antibiotic resistance and resilience in adverse environments compared to the planktonic state (Yan and Bassler 2019). Biofilms formed by pathogenic bacteria pose significant challenges to humans, particularly in chronic infections (Barman et al. 2024). However, research on biofilms formed by beneficial microorganisms remains limited and underexplored.

It is well known that there are a number of challenges when applying probiotics clinically due to their high sensitivity to the harsh gastrointestinal (GI) conditions (Centurion et al. 2021; Li et al. 2024). Encapsulating probiotics in carriers that offer protective and targeted delivery capabilities can enhance their tolerance to harsh external and gastrointestinal environments (Luo et al. 2022). Biofilm has been considered an effective method for encapsulating and delivering probiotics. Compared to other encapsulation methods, such as oil gels and hydrogels, biofilm encapsulation appears to be safer and simpler, as it does not introduce exogenous substances (Li et al. 2024; Luo et al. 2022).

According to published literature, delivering probiotics in the state of biofilm can significantly improve their adhesion ability and maintain high viability throughout the gastrointestinal tract (Al‐Hadidi et al. 2021; Gao et al. 2022; Hu et al. 2022). Liu et al. reported that biofilm cells of *Lactiplantibacillus paraplantarum* LR‐1 and *Lactobacillus paraplantarum* L‐ZS9 exhibited higher immunomodulatory activity than their planktonic cells (Liu et al. 2023, 2021). Liu et al. further observed that amino acid and carbohydrate metabolism in the biofilm state of *L. plantarum* was more active than in its planktonic state (Liu et al. 2021). The biofilms of *Lactobacillus* and *Bifidobacterium* activated dendritic cells through Toll‐like receptor 2 signaling, inhibiting tumor growth and enhancing the efficacy of chemotherapy and immunotherapy (Han et al. 2021). *Lactobacillus reuteri* in biofilm state reduced the severity and incidence of *Clostridium difficile* infection (Shelby et al. 2020). Additionally, Sun et al. employed transcriptome sequencing to compare the differentially expressed genes (DEGs) between biofilm and planktonic states of *Lactobacillus plantarum* J26, finding that the DEGs were primarily enriched in adhesion, pyrimidine metabolism, glycerol metabolism, stress response, and quorum‐sensing pathways (Sun et al. 2020). A comprehensive study on the characteristics of probiotic biofilms is expected to promote the widespread clinical application of probiotics.

Many studies have proven that *Lactobacillus* and *Bifidobacterium* have multiple health benefits and are also the most commonly used microorganisms for the commercialization of probiotic products (Bober et al. 2018; Hojjati et al. 2020; Turroni et al. 2014). Limited research on the biofilm properties of *Lactobacillus* and *Bifidobacterium* probiotics indicates the need for further investigation (Silva et al. 2020). This study selected strains from the two genera to compare the probiotic traits of their biofilm and planktonic states. *Ligilactobacillus salivarius* Li01, reported to have potential therapeutic effects on gastrointestinal and liver‐related conditions, was selected due to its strong biofilm‐forming ability demonstrated in preliminary screening (Fei et al. 2022; Qiu et al. 2022; Zhuge et al. 2020). Similarly, two *Bifidobacterium* strains, *Bifidobacterium longum* , and *Bifidobacterium pseudocatenulatum* , were chosen for their strong biofilm formation ability identified during pre‐screening. In the present study, the gastrointestinal tolerance and intestinal adhesion abilities of the three bacterial strains in both biofilm and planktonic states were measured to explore the advantages of biofilm more comprehensively. Additionally, transcriptomic analysis was used to explore the tolerance mechanisms of Li01 biofilm in harsh environments.

## 2 Materials and Methods

### 2.1 Bacterial Strains, Caco‐2 Cells and Culture Conditions

*Ligilactobacillus salivarius* Li01 was obtained from the First Affiliated Hospital of Zhejiang University School of Medicine and cultured in MRS broth (Oxoid, Basingstoke, UK) at 37°C in a vinyl anaerobic chamber (Coy Laboratories, USA). *Bifidobacterium longum* and *Bifidobacterium pseudocatenulatum* were isolated from healthy human feces and cultured in TPY broth (Hopebio, China) at 37°C in a vinyl anaerobic chamber (Coy Laboratories, USA). Caco‐2 cells were cultured in Dulbecco's Modified Eagle medium (DMEM, Gibco, China), supplemented with 10% heat‐inactivated fetal bovine serum (FBS, Gibco, China) and 1% penicillin‐streptomycin (Gibco, China). The cells were maintained at 37°C in a 5% CO_2_ atmosphere.

### 2.2 Biofilm Formation and Growth

A crystal violet staining assay was performed to evaluate bacterial biofilm formation and growth (Zhang, Meng, et al. 2022). The bacterial suspension was diluted to 2% (v/v) in fresh MRS or TPY medium and added into a 96‐well cell culture plate at 200 μL per well. The plates were incubated at 37°C for 6, 12, 24, 36, 48, 54, 60, and 72 h, after which the medium was carefully removed and the wells were washed three times with PBS. After drying, the biofilms were fixed in methanol for 10 min, and the methanol was discarded. The wells were stained with 200 μL of crystal violet per well for 10 min and washed three times with PBS. After drying again, 33% glacial acetic acid was added to each well, and the plate was incubated at room temperature for 30 min. The optical density at 570 nm (OD_570_) was then measured using a microplate reader. The MRS and TPY medium were used as the negative control. The ODc value was calculated as three times the standard deviation added to the average absorbance of the control group at 570 nm. OD ≤ ODc, 2*ODc < OD ≤ 4*ODc, and OD > 4*ODc were interpreted as indicating no biofilm formation, moderate biofilm formation, and strong biofilm formation ability, respectively.

### 2.3 External Factors Impacting Biofilm Formation

To investigate the effects of different pH (3, 4, 5, 6, 7, 8, and 9) and bile salts (0, 0.05%, 0.1%, 0.2%, and 0.3%, w/v) on biofilm formation in the culture medium, a crystal violet staining assay was conducted on the three bacterial strains cultured for 48 h, following the previously described method.

### 2.4 Morphological Observation of Biofilm

Cell crawling slices were placed in 12‐well plates containing 4 mL of fresh culture medium. Bacterial strains were inoculated at 2% (v/v) and incubated anaerobically at 37°C for 48 h. After incubation, the crawling slices were removed and washed three times with PBS to remove planktonic bacteria. The slices were then fixed in 2.5% glutaraldehyde solution at 4°C in the dark for 16 h. To prepare planktonic bacterial cells for electron microscopy, the cell pellet was collected by centrifugation and resuspended in 1 mL of glutaraldehyde fixative. After fixation, the slices and cell pellet were washed three times with PBS and treated with 1% osmium tetroxide for 1 h. The samples were washed three times with PBS. Subsequently, the samples were dehydrated using a gradient ethanol series (30%, 50%, 70%, 80%, 90%, 95%, and 100%). The dehydrated samples were dried with a Hitachi HCP‐2 critical point dryer and coated for observation. Finally, the sample morphology was imaged with a Hitachi SU‐8010 scanning electron microscope (Hu et al. 2023).

### 2.5 Preparation of Planktonic and Biofilm Strains

Planktonic strains were cultured anaerobically at 37°C and 150 rpm. Biofilm cells were grown on 12‐well cell culture plates. Following anaerobic incubation of the three bacterial strains, the suspension was centrifuged to discard the supernatant. The pellet was resuspended in PBS, and 2% (v/v) of the suspension was inoculated into MRS or TPY medium. Then, 2 mL was added per well in 12‐well polystyrene microplates. The plates were incubated anaerobically at 37°C. After biofilm formation, the medium was carefully removed from the wells, which were then washed three times with PBS to eliminate planktonic bacteria. Biofilm cells were collected using cell scrapers, washed twice with PBS, and resuspended in PBS. Planktonic bacterial cells were similarly centrifuged and resuspended in PBS.

### 2.6 In Vitro Gastrointestinal Tolerance

The gastrointestinal tolerance simulation experiment was adapted from previous studies (Qiu et al. 2024; Shi et al. 2022; Yao et al. 2017). Planktonic and biofilm strains were prepared using the method described above. Briefly, the bacterial solution was mixed with simulated gastric fluid (SGF, pH 2.0) or simulated intestinal fluid (SIF, 0.15% g/v bile salts) (Source Leaf, Shanghai, China) at a 1:9 volume ratio. The mixture was incubated anaerobically on a shaker (100 rpm) at 37°C for 30, 60, 90, and 120 min. Biofilm cells were treated with ultrasound for 3 min (40 kHz, 50 W, KQ‐50B) to release biofilm cells. Gradient dilution plate counting was performed to quantify viable bacteria.

### 2.7 Surface Hydrophobicity Analysis

Planktonic and biofilm cells were prepared using the method described above. The bacterial suspension was adjusted to an OD600 of 0.4 (A0). 1 mL of xylene was added to 3 mL of bacterial suspension, and the mixture was vortexed for 1 min. The mixture was incubated at 37°C for 20 min, and the OD600 of the aqueous phase (A1) was measured. The hydrophobicity percentage was calculated using the formula: Hydrophobicity (%) = (1 – A1/A0) × 100 (Berkes et al. 2020; Echresh et al. 2024).

### 2.8 Adhesion Assay

Based on the methods of Qiu and Zhang, the adhesion assay was performed with minor modifications (Berkes et al. 2020; Qiu et al. 2024; Zhang, Meng, et al. 2022). Caco‐2 cells were seeded into 12‐well plates and incubated at 37°C in a 5% CO_2_ atmosphere. The culture medium was refreshed every other day until a confluent monolayer formed. The cells were then washed once with DPBS. Each well was treated with 800 μL of bacterial suspension, prepared as described above, and incubated for 1.5 h at 37°C in a 5% CO_2_ atmosphere. Non‐adherent bacteria were removed by washing the wells twice with PBS after incubation. The cells were lysed with 1% Triton X‐100 for 10 min, and adherent bacteria were quantified using the gradient dilution plate method. The adhesion capacity of bacteria was calculated using the following formula:

$$\begin{array}{c} \text{Adhesion rate}\;\left(\%\right)\\ =\frac{\;\text{The number of bacteria adhering to cells}\;\left(\mathrm{CFU}/\mathrm{mL}\right)}{\text{The number of initial bacteria}\;\left(\mathrm{CFU}/\mathrm{mL}\right)}\times 100 \end{array}$$

### 2.9 Zeta Potential Analysis

Planktonic and biofilm cells were prepared using the method described above. The zeta potential of planktonic bacterial cells and biofilm cells was measured using a particle electrophoresis instrument (Zetasizer Nano‐ZS, Malvern Panalytical, Malvern, UK) (Hojjati et al. 2020; Qiu et al. 2024).

### 2.10 EPS Extraction of Biofilm

The preparation of biofilm was performed by using the method described above. Biofilm suspensions were treated with ultrasound for 3 min (40 kHz, 50 W), followed by centrifugation at 10,000 rpm and 4°C for 25 min to separate EPSs from the cells (Homero et al. 2021). The supernatant obtained after centrifugation was divided into three equal volumes for the analysis of EPS components (polysaccharides, proteins, and DNA).

### 2.11 Extraction and Quantification of Polysaccharides

The EPS‐containing supernatant was mixed with ethanol at a 1:3 volume ratio and refrigerated at 4°C for 18 h to precipitate polysaccharides. The precipitated polysaccharides were collected by centrifugation and quantified using the phenol‐sulfuric acid method (Mathivanan et al. 2023). The polysaccharide solution, 5% (w/v) phenol solution, and concentrated H_2_SO_4_ were mixed at a volume ratio of 1:1:5. The mixture was incubated in darkness at room temperature for 30 min. The optical density at 490 nm (OD_490_) was measured.

### 2.12 Protein and DNA Quantification of EPS

Extracellular protein content was quantified using the Enhanced BCA Protein Assay Kit (Beyotime, China). DNA was extracted using the DNeasy PowerSoil Pro Kit (Qiagen, Germany) and quantified with a NanoDrop 2000 micro‐ultraviolet spectrophotometer (Thermo Scientific, USA) (Lin et al. 2023).

### 2.13 Transcription Assays

The total RNA of *Ligilactobacillus salivarius* Li01 planktonic cells and biofilm cells was extracted using TRIzol Reagent or the RNeasy Mini Kit (Qiagen), quantified and assessed for quality using the Agilent 2100/2200 Bioanalyzer, NanoDrop, and 1% agarose gel. RNA (300–500 ng) was hybridized with a single‐stranded DNA probe targeting rRNA, followed by digestion of the rRNA and probe. The remaining RNA was purified with RNA Clean Beads, fragmented with divalent cations, and reverse‐transcribed to cDNA using random primers. During second‐strand synthesis, dUTP incorporation enabled 5′ phosphorylation and 3′ adenylation. Sequencing adapters were added to both ends of the cDNA, which was size‐selected and purified with DNA Clean Beads. PCR amplification was performed with UDG to remove the second strand containing dUTP, and the library was amplified using P5 and P7 primers. Quality control checks were performed, and the libraries, each tagged with a unique index, were pooled and subjected to PE150 sequencing on the Illumina Novaseq6000 or MGI2000, following manufacturer guidelines (Kechin et al. 2017; Trapnell et al. 2010).

### 2.14 Transcriptome Sequencing Data Analysis

To ensure high‐quality data, raw sequencing reads in FASTQ format were processed using Cutadapt (v1.9.1) to remove technical sequences, such as adapters, PCR primers, or fragments thereof, as well as bases with a phred quality score below 20. The parameters used included an error rate of 0.1, a minimum adapter overlap of 1 bp, a minimum sequence length of 75 bp, and a maximum N proportion of 0.1. Next, the reference genome of Li01 was indexed using Bowtie2 (v2.2.6), and clean reads were aligned to the reference genome using the same software. Gene expression levels were then estimated with HTSeq (v0.6.1p1), using transcripts converted from GFF annotation files to FASTA format and indexed as a reference. Differential expression analysis was performed using the DESeq2 Bioconductor package, which applies a negative binomial distribution model. Genes with an adjusted *p*‐value (*p*adj) < 0.05, corrected using Benjamini and Hochberg's method to control the false discovery rate, were identified as differentially expressed. For functional enrichment, GOSeq (v1.34.1) was used to identify significantly enriched Gene Ontology (GO) terms (*p* < 0.05). KEGG (Kyoto Encyclopedia of Genes and Genomes) is a collection of databases dealing with genomes, biological pathways, diseases, drugs, and chemical substances (http://en.wikipedia.org/wiki/KEGG). We used scripts in house to enrich significant differential expression gene in KEGG pathways.

### 2.15 Statistical Analysis

Each experiment was repeated three times, and the results were shown as mean ± standard deviation (SD). *p < 0.05, **p < 0.01, ***p < 0.001. We used Student's *t*‐test or One‐way ANOVA test for statistical analysis, with GraphPad Prism (version 9.4.1).

## 3 Results and Discussion

### 3.1 Biofilm Growth and the Effects of pH and Bile Salts on Biofilm Formation

**FIGURE 1:**
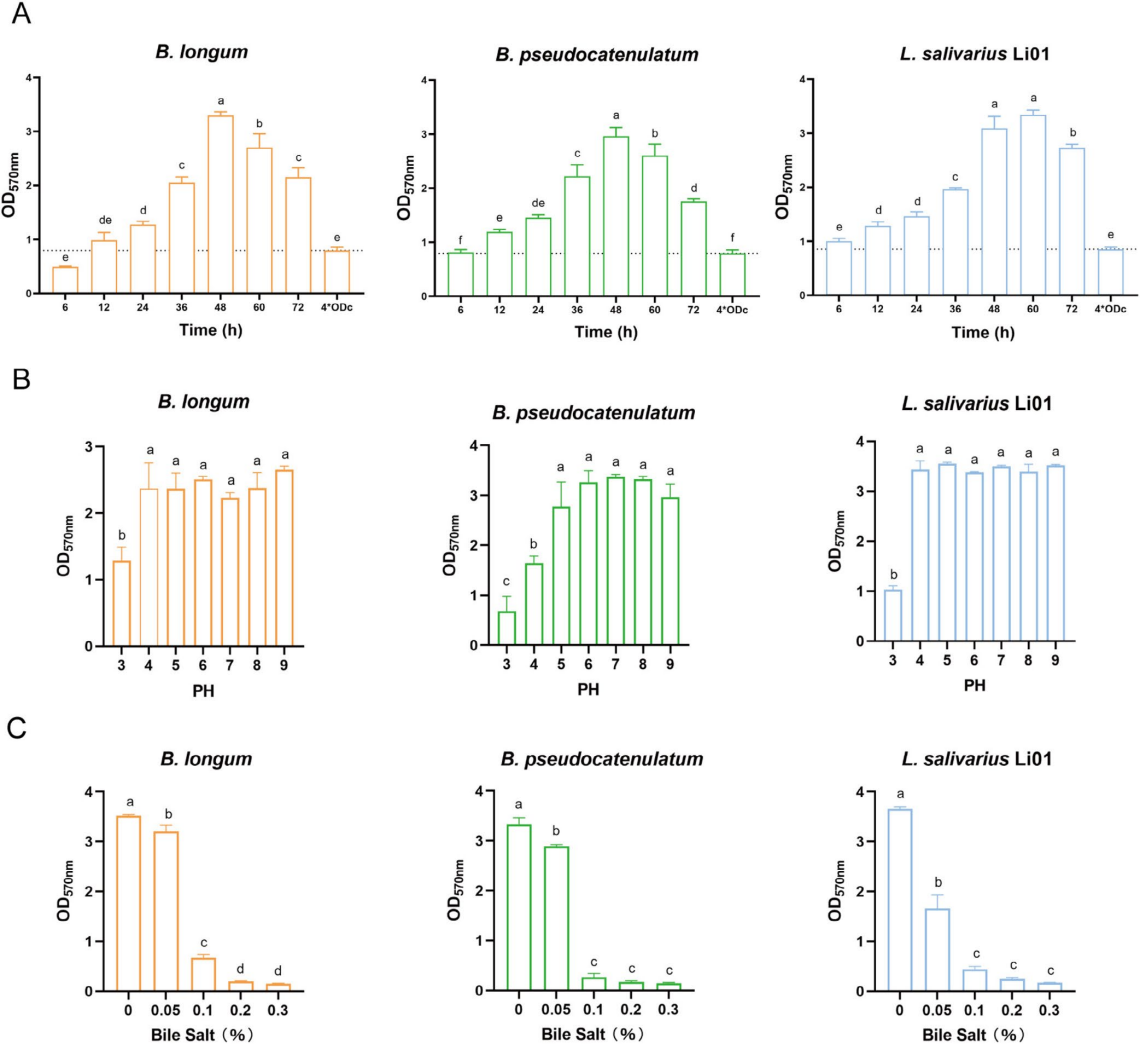
(A) Effect of cultivation time on biofilm growth. (B) Effect of different PH on biofilm formation. (C) Effect of different bile salt concentration (%) on biofilm formation. Different letters indicate significant differences between groups (*p* < 0.05).

Biofilm formation is a dynamic, multifactorial process influenced by environmental conditions and bacterial intrinsic properties, including incubation time, nutrient availability, temperature, pH, carrier surface characteristics, and strain‐specific traits (Guzmán‐Soto et al. 2021; Salas‐Jara et al. 2016). As shown in Figure 1A, after 12 h of incubation, *B. longum* , *B. pseudocatenulatum* , and *L. salivarius* Li01 exhibited OD values exceeding 4ODc, indicating strong biofilm‐forming ability. Bacterial biofilm development generally progresses through four distinct phases: initial attachment, early biofilm architecture formation (microcolony development), maturation, and dispersion (Kilic and Bali 2023). The biofilm biomass of Bifidobacterium strains peaked at 48 h before declining. The biofilm biomass of *L. salivarius* Li01 continuously increased until 60 h, after which it decreased. Consistent with previous studies, our results showed that the biofilm biomass increased continuously, reached its maximum, and then gradually decreased due to biofilm dispersal (Ding et al. 2019; Kilic and Bali 2023). Biofilm dispersion may be associated with nutrient depletion caused by prolonged incubation (Guzmán‐Soto et al. 2021). Additionally, limited space within microplate wells may constrain the growth of biofilm‐forming microorganisms. This dynamic development of biofilm highlights its strategies for survival in resource‐limited environments and facilitates the colonization of new niches (Kilic and Bali 2023).

In the human gastrointestinal tract, microorganisms face significant challenges from low pH and high bile salt concentrations. As shown in Figure 1B, the biofilm biomass of the three strains was markedly reduced at pH 3 compared to other pH conditions. Acid stress disrupts biofilm aggregation by reducing the levels of essential components in the biofilm matrix (Li et al. 2023). The acid stress alters the matrix's electrostatic properties and structural morphology, further affecting biofilm stability (di Biase et al. 2022). However, their biofilm production remained unaffected when cultivated in alkaline cultivation environments. Additionally, the biofilm biomass of the three strains decreased sharply at 0.1% bile salt concentration and remained low as the bile salt concentration increased further (Figure 1C). These findings highlighted the sensitivity of probiotic biofilm formation to environmental factors, particularly low pH and high bile salt concentrations in the gastrointestinal tract.

### 3.2 Morphological Observations of Biofilm Cells and Planktonic Cells

**FIGURE 2:**
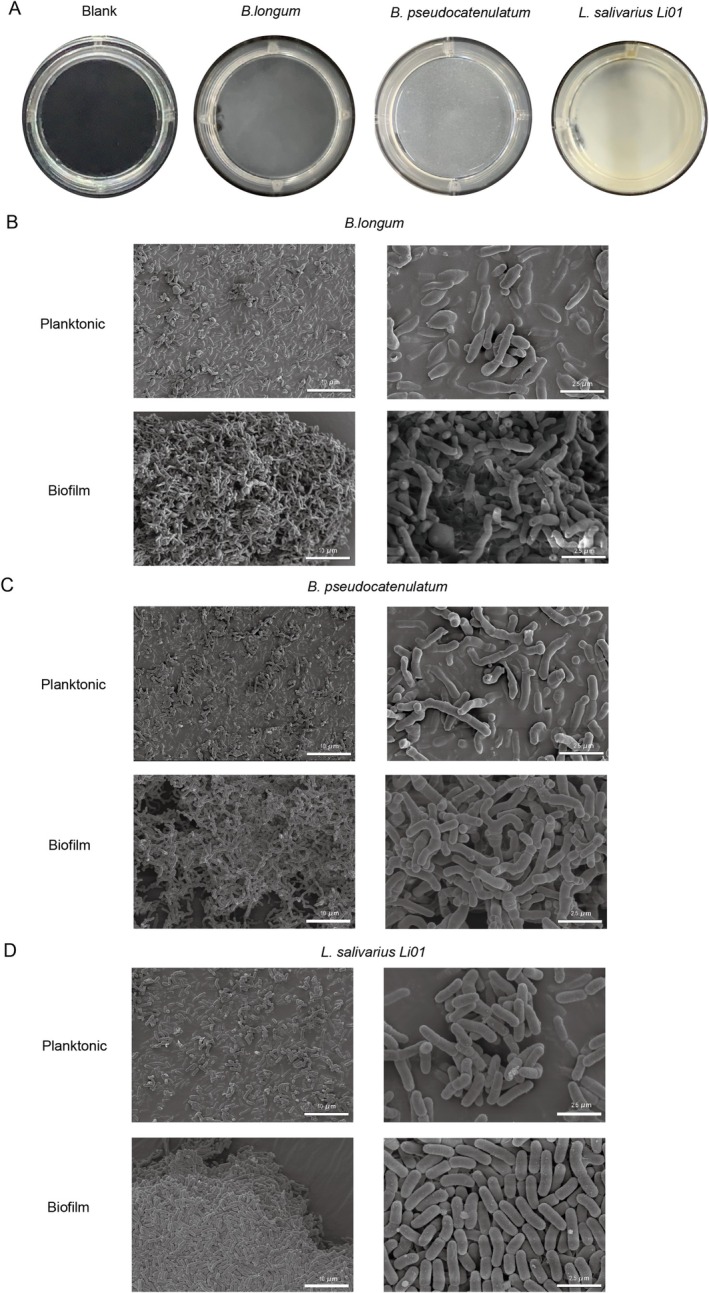
(A) Visual observation of biofilms incubated on 12 well plates for 48 h. (B–D) Scanning electron micrographs of biofilm and planktonic states of three strains.

Microorganisms in the human gastrointestinal tract primarily exist as biofilms (Kilic and Bali 2023; Salas‐Jara et al. 2016). The biofilm morphology of the three strains on 12‐well plates, as observed macroscopically, was shown in Figure 2A. The biofilms uniformly cover the well bottoms, adhering firmly and resisting removal by PBS washing. Notably, the biofilm formed by *L. salivarius* Li01 was thicker and denser than those of the other two strains. To further investigate the microstructure of biofilms, electron microscopy was conducted (Figure 2B,C). Overall, these biofilms displayed a tightly packed, multilayered, and three‐dimensionally organized structure. In contrast, electron microscopy of planktonic bacteria revealed that the cells were dispersed, with clearly visible morphology, and the phenomenon of mutual aggregation between bacterial cells was not obvious. Consistent with the macroscopic observations, the biofilm of *L. salivarius* Li01 displayed bacterial cells in exceptionally close contact with one another and a distinct multilayered structure compared to the biofilms of the other two strains. The biofilm structure could enhance quorum sensing among microorganisms and improve their tolerance to harsh environmental conditions (Li et al. 2023).

### 3.3 Viability of Three Strains in the Planktonic and Biofilm States Under Simulated Gastrointestinal Conditions

**FIGURE 3:**
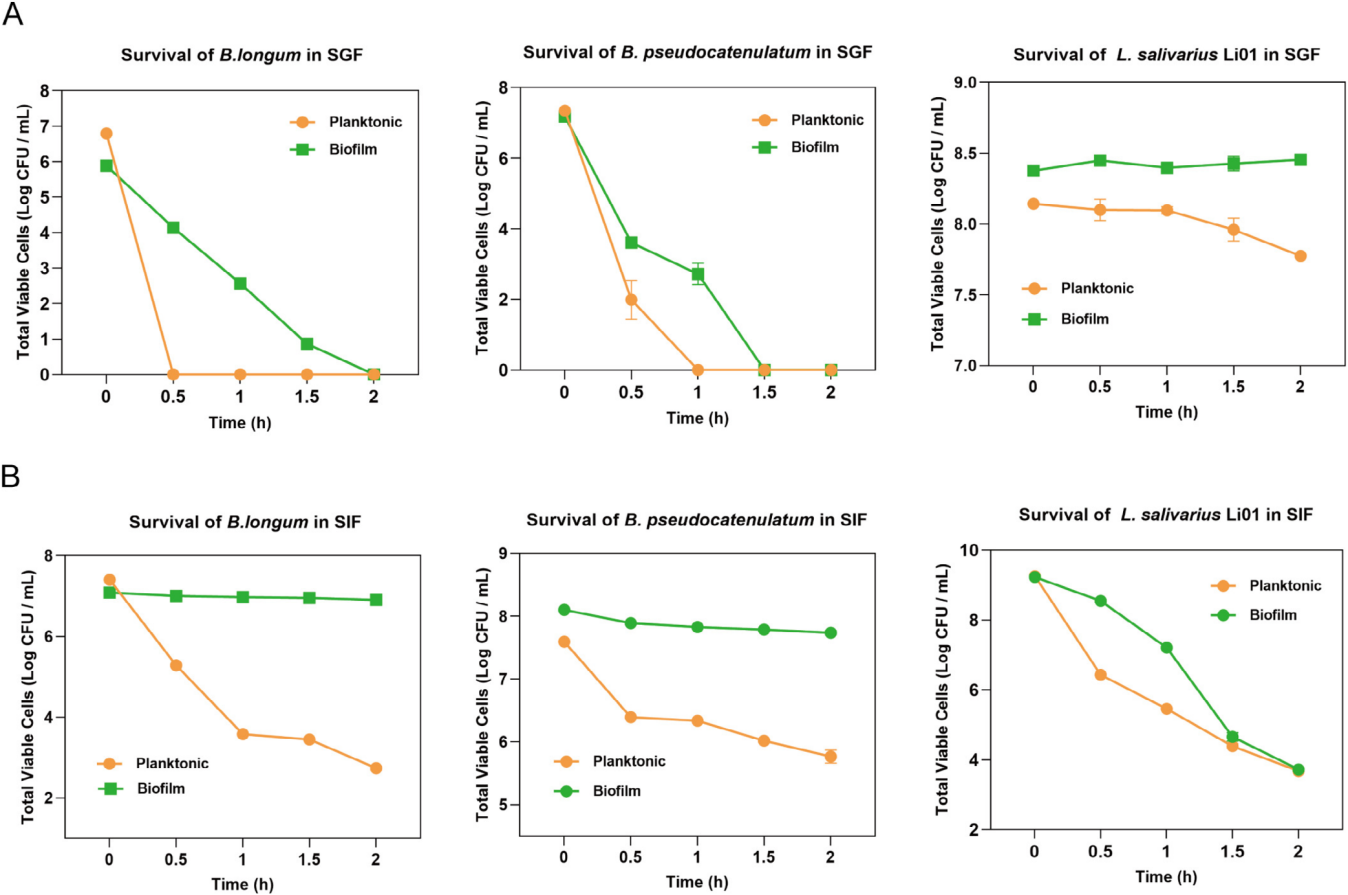
Variations in the number of viable bacteria in biofilm and planktonic states in simulated gastric juice (A) and simulated intestinal fluids (B).

Delivering sufficient probiotics to the gut can directly improve intestinal health and overall well‐being. However, gastric acid, bile salts, and digestive enzymes in the gastrointestinal tract could damage or inactivate probiotics, reducing their effectiveness (Xu, Guo, et al. 2024). Biofilms could serve as delivery vehicles by encapsulating probiotics, offering enhanced protection from harsh gastrointestinal conditions (Salas‐Jara et al. 2016). This study examined the tolerance of three bacterial strains in planktonic and biofilm states to simulated gastrointestinal fluids, as shown in Figure 3. In simulated gastric fluid (SGF) digestion (Figure 3A), the biofilm state greatly improved bacterial survival compared to the planktonic state. For *B. longum* , planktonic cells were completely inactivated within 30 min, while biofilm cells remained viable cells detectable for up to 1.5 h. Similarly, *B. pseudocatenulatum* biofilms reduced cell loss, retaining 2.72 log_10_ CFU/mL viable cells after 1 h of SGF digestion, compared to complete inactivation of planktonic cells. For *L. salivarius* Li01, planktonic cells exhibited strong tolerance to gastric fluid with minimal reduction, whereas its biofilm state maintained near‐constant viability over 2 h. In simulated intestinal fluid (SIF) digestion (Figure 3B), the biofilm state also greatly improved bacterial survival. *B. longum* biofilms maintained almost constant viability over 2 h of intestinal fluid digestion, whereas planktonic cells exhibited a 4.67 log_10_ CFU/mL reduction after 2 h. *B. pseudocatenulatum* biofilms had lower viability loss (0.36 log_10_ CFU/mL) after 2 h, compared to a 1.83 log_10_ CFU/mL reduction in planktonic cells. During the first hour of SIF digestion, the biofilm state of *L. salivarius* Li01 exhibited greater resistance, with smaller reductions in viable cell counts than the planktonic state. However, after 1.5–2 h, viable counts in both states converged, likely due to bile salt‐induced biofilm damage.

Overall, biofilm cells improved probiotics' resistance to digestive fluids, leading to higher survival rates during gastrointestinal digestion compared to planktonic bacteria, consistent with findings from previous studies (Li et al. 2023; Zhang, Gu, et al. 2022). The enhanced survival rate observed during gastrointestinal digestion following biofilm formation is likely attributable to the permeability barrier created by the biofilm. Due to the three‐dimensional structure of biofilms, external substances may need to pass through the matrix channels before interacting with the bacteria within the biofilm. During biofilm growth, the bacteria produced a large amount of EPSs, which surrounded the cells. These exopolysaccharides restricted the entry of external agents, thereby increasing bacterial resistance to gastrointestinal digestion (Karygianni et al. 2020; Toledo‐Arana et al. 2001). Furthermore, the high cell density within biofilms facilitated synergistic interactions, enhancing their resistance to unfavorable environments (Yao et al. 2022). Bacteria in the biofilm's deeper layers were inactive, which contributed to their resilience to environmental stress (Raad et al. 1998).

### 3.4 Comparison of Adhesion Ability and Cell Surface Properties Between Planktonic Cells and Biofilm Cells

**FIGURE 4:**
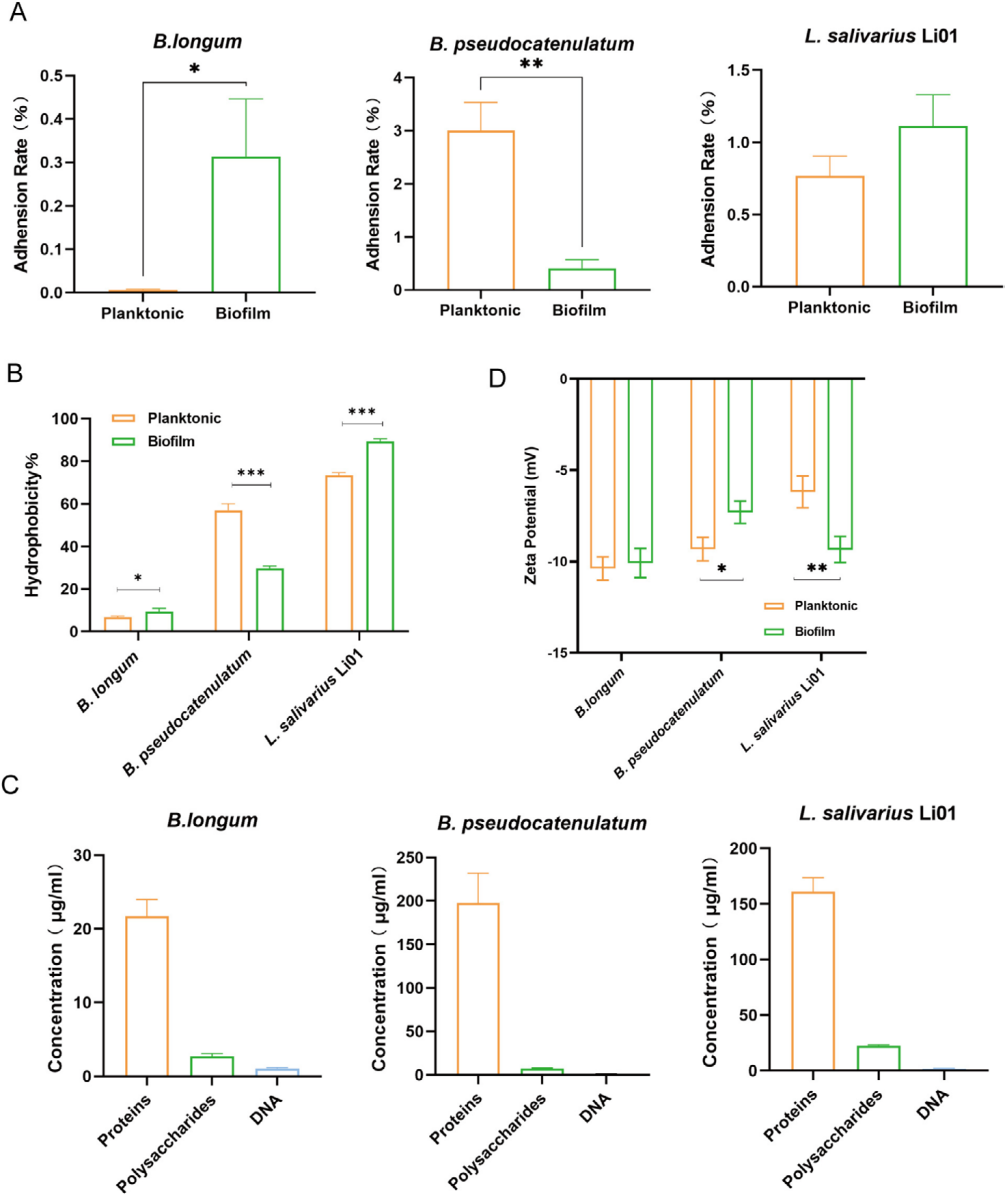
(A) Comparison of adhesion rates on Caco‐2 cell monolayers between planktonic and biofilm states. Measurement of hydrophobicity (B) and Zeta potential (D) in planktonic and biofilm states. (C) Quantification of the main components of EPS in the biofilm.

The high adherence of probiotics to the intestinal epithelium is crucial for their colonization and rapid proliferation within the gut (Krausova et al. 2019). We developed a Caco‐2 monolayer cell model to assess the intestinal adhesion capacities of the three strains (Figure 4A). *B. longum* exhibited a significantly higher adhesion rate to Caco‐2 monolayers in the biofilm state than in the planktonic state (*p* < 0.05). Additionally, *L. Salivarius* Li01 exhibited a 1.45‐fold higher adhesion rate to Caco‐2 monolayers in the biofilm state than in the planktonic state. However, the biofilm state of *B. pseudocatenulatum* showed a significantly lower adhesion rate to Caco‐2 monolayers compared to the planktonic state (*p* < 0.01). Previous studies have generally shown that the biofilm state of probiotics enhances adhesion to intestinal cells compared to the planktonic state (Berkes et al. 2020; Li et al. 2023; Zhang, Meng, et al. 2022).

Probiotic adhesion to intestinal cells can be classified into two types: non‐specific adhesion and receptor‐specific adhesion. Among them, non‐specific adhesion was primarily driven by hydrophobic interactions and electrostatic forces (Monteagudo‐Mera et al. 2019). Strains with higher hydrophobicity generally exhibit stronger adhesion to intestinal epithelial cells (Yang et al. 2023). Therefore, surface hydrophobicity may be an important factor in determining the adhesion capabilities of probiotics. As shown in Figure 4B, the results showed that the hydrophobicity of the three strains was positively correlated with their adhesion to intestinal epithelial cells. The hydrophobicity of *B. longum* and *L. salivarius* Li01 was significantly higher in the biofilm state than in the planktonic state (*p* < 0.05). However, the hydrophobicity of *B. pseudocatenulatum* in the biofilm state was significantly lower than that in the planktonic state (*p* < 0.001), which explained its lower adhesion rate in the biofilm state. Most studies have also suggested that probiotics in the biofilm state exhibit stronger intestinal adhesion than those in the planktonic state, promoting better proliferation in the gut (Berkes et al. 2020; Li et al. 2023; Zhang, Meng, et al. 2022). The present work suggested that the biofilm state does not always enhance probiotic adhesion to the intestine. This phenomenon may be influenced by factors such as strain‐specific differences, hydrophobicity, and the composition of EPSs. For example, a study by Tahoun revealed that extracellular polysaccharides inhibited the intestinal adhesion capacity of *Bifidobacterium longum* 105‐A, while research by Dertli demonstrated a similar effect on *Lactobacillus johnsonii* FI9785 (Dertli et al. 2015; Tahoun et al. 2017).

Biofilms are structured bacterial communities consisting of bacteria immobilized on a surface, along with self‐produced proteins, DNA, and polysaccharides. These EPSs could alter cell surface physicochemical properties, including hydrophobicity and surface charge, thereby influencing bacterial adhesion and colonization (Deepika et al. 2009; Dertli et al. 2015; Harimawan and Ting 2016). The interaction between EPS and intestinal mucins also plays a crucial role in bacterial adhesion (Karygianni et al. 2020). In this study, the contents of protein, polysaccharide, and DNA of the EPS from the biofilms of the three probiotic strains were quantitatively analyzed (Figure 4C). The obtained results showed that proteins were the dominant component of the EPS in all strains, while polysaccharides and DNA contributed minimally. Savijoki et al. identified and compared the cell surface‐associated proteins of *Lactobacillus rhamnosus* GG in planktonic and biofilm states, finding that the expression of adhesion‐associated proteins was significantly upregulated (Savijoki et al. 2019). However, the high protein content in *B. pseudocatenulatum* EPS (approximately 200 μg/mL) may cause steric hindrance, limiting the exposure of adhesion molecules to the intestinal surface, thus reducing its adhesion capacity. The results above suggested that the protein of EPS might be the critical factor that influenced the intestinal adhesion capacity of all three probiotic strains. Also, the role of EPSs in intestinal adhesion during biofilm development needs further investigation.

The surface zeta potentials of the three strains were analyzed to evaluate the effect of EPS on the surface charge of biofilm cells. As shown in Figure 4D, the zeta potential of *B. longum* showed no significant difference between the planktonic and biofilm states, indicating that the increased biofilm adhesion of *B. longum* was influenced by other factors, such as surface hydrophobicity and the components of EPS. The biofilm cells of *B. pseudocatenulatum* exhibited a higher zeta potential than its planktonic cells (*p* < 0.05), which would theoretically enhance its ability to adhere to intestinal cells via electrostatic interactions. However, the results showed that *B. pseudocatenulatum* biofilm cells had lower hydrophobicity and adhesion ability compared to its planktonic cells. This may be attributed to the high content and relatively low negative charge of the hydrophilic substances in its biofilm's EPS. The biofilm cells of *L. salivarius* Li01 had a significantly lower zeta potential than the planktonic cells (*p* < 0.01), which would theoretically lead to decreased adhesion to the gut; yet, the results indicated that both adhesion and hydrophobicity of Li01 biofilm actually increased. This might be caused by the strong negative charge and high concentration of hydrophobic substances in the EPS of Li01 biofilm. Therefore, consistent with Dertli's findings (Dertli et al. 2015), the zeta potential measurements in the present study showed that EPS influenced the surface charge of probiotics.

Overall, probiotic adhesion to intestinal epithelial cells is governed by a complex interplay of factors, including surface hydrophobicity, the composition and structure of EPSs, and bacterial surface charge, with each factor contributing differently depending on the specific strain.

### 3.5 Screening and Clustering Analysis of Differentially Expressed Genes

**FIGURE 5:**
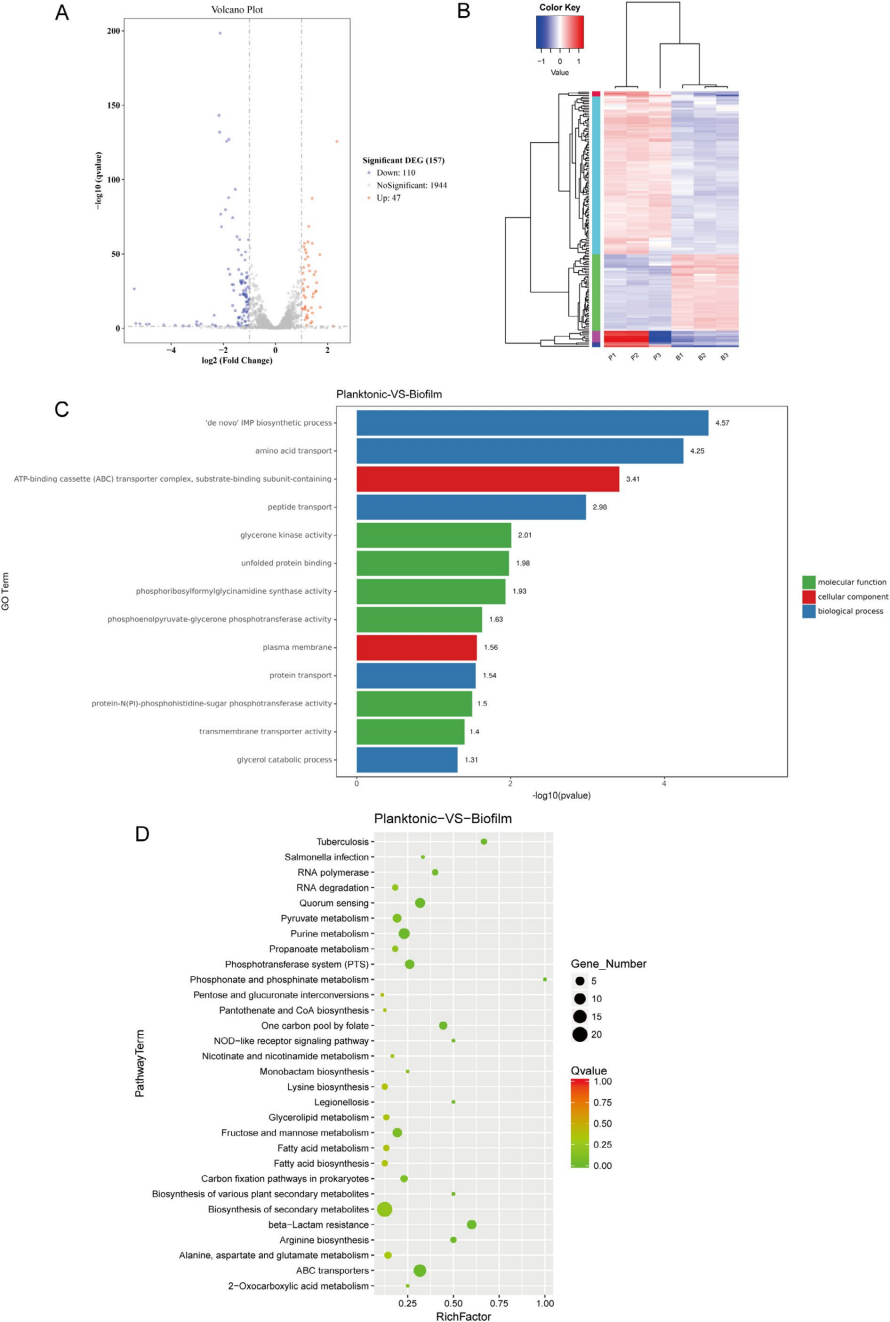
(A) Volcano plot of differentially expressed genes between the planktonic and biofilm states of *L. salivarius* Li01. Differentially expressed genes were visualized as red dots for up‐regulated genes and blue dots for down‐regulated genes. The *X*‐axis represented the fold change in gene expression, and the Y‐axis reflected the statistical significance of these changes. (B) Cluster analysis of differentially expressed gene between the planktonic and biofilm states of *L. salivarius* Li01. Clustering was conducted using log_10_(FPKM + 1) values. Red indicated highly expressed genes, while blue represented lowly expressed genes, with the gradient from blue to red denoting increasing expression levels. (C) Bar plot of GO enrichment analysis based on *p*‐values. The vertical axis represented the enriched GO terms, and the horizontal axis displayed −log10(*p*‐value), where larger values denoted higher enrichment significance. (D) Scatter plot of pathway enrichment analysis. The vertical axis listed pathway names, and the horizontal axis represented the Rich factor. The dot size reflected the number of differentially expressed genes per pathway, and the dot color indicated the *Q*‐value. Higher Rich factors and smaller *Q*‐values represented greater enrichment significance.

Several studies have demonstrated that *Lactobacillus salivarius* Li01 holds significant potential for treating various diseases (Fei et al. 2022; Xu, Qiu, et al. 2024; Yang et al. 2020; Zhuge et al. 2021). Our findings revealed that *Lactobacillus salivarius* Li01 in the biofilm state exhibited higher adhesion to intestinal epithelial cells and greater gastrointestinal tolerance compared to the planktonic state. The transcriptomic analysis of *L. sialicum* Li01 was carried out to investigate the molecular mechanisms underlying the effects of biofilm formation on physiological characteristics. Sequencing data were filtered using a threshold of ≥ twofold change in gene expression and *q*‐value (FDR, *p*adj) ≤ 0.05. A total of 157 DEGs were identified in the biofilm state of *L. salivarius* Li01, including 110 down‐regulated and 47 up‐regulated genes, compared to its planktonic state (Figure 5A). Hierarchical clustering analysis of the DEGs, based on FPKM values, demonstrated significant differences in gene expression between the planktonic and biofilm states of *L. salivarius* Li01 (Figure 5B). The results highlighted significant differences in gene expression between the planktonic and biofilm states of *L. salivarius* Li01, emphasizing the complex and adaptive nature of biofilm formation.

### 3.6 GO and KEGG Enrichment Analysis of Differentially Expressed Genes

To further explore the biological functions and molecular pathways of the DEGs, we performed GO and Kyoto Encyclopedia of Genes and Genomes (KEGG) enrichment analyses. GO enrichment analysis was performed to identify biological functions significantly associated with the DEGs, which were categorized into molecular functions, cellular components, and biological processes. As shown in Figure 5C, in the biological process category, DEGs were primarily enriched in the de novo IMP biosynthetic process, amino acid transport, peptide transport, and protein transport. In the cellular component category, DEGs were primarily associated with the ATP‐binding cassette (ABC) transporter complex, substrate‐binding subunit, and plasma membrane. In the molecular function category, DEGs were enriched in glycerone kinase activity, unfolded protein binding, phosphoribosylformylglycinamidine synthase activity, and phosphoenolpyruvate‐glycerone phosphotransferase activity.

Genes in organisms work together to perform biological functions. Pathway enrichment analysis highlighted key biochemical, metabolic, and signaling pathways associated with DEGs. KEGG enrichment analysis of DEGs was performed, and the top 30 significantly enriched pathways were selected for presentation in Figure 5D. Table 1 showed all pathways that were significantly enriched in these 30 pathways with *q*‐values < 0.05. As shown in Figure 5D and Table 1, DEGs were primarily enriched in pathways such as ABC transporters, One carbon pool by folate, Quorum sensing, Purine metabolism, Arginine biosynthesis, Phosphotransferase system (PTS), RNA polymerase, and NOD‐like receptor signaling pathway. In the planktonic state, bacteria rapidly proliferate, relying on efficient transport systems to acquire nutrients and sustain high metabolic activity. In contrast, biofilm cells enter a “low‐metabolism mode” to ensure long‐term survival and stability (Klopper et al. 2020; Zhao et al. 2023). ABC transporters, commonly found in bacteria, function as active transmembrane transport systems powered by ATP hydrolysis. They perform various physiological roles, such as nutrient acquisition, signal molecule transport, and toxin efflux (Davidson and Chen 2004). Additionally, ABC transporters are closely associated with quorum sensing (QS), a key regulatory mechanism for biofilm formation and maintenance (Solano et al. 2014). As indicated in Table 1, the DEGs involved in the ABC transporters and QS pathway were mainly associated with ABC transporter functions. The downregulation of amino acid and peptide transport‐related genes suggested that biofilm‐associated bacteria reduce their dependence on external amino acids and peptides. Bacterial metabolic activity within biofilms may produce waste products or by‐products, including organic acids and toxic metabolites. The upregulation of DMT transporter genes in the QS pathway suggested that these transporters assisted in removing such substances, contributing to biofilm stability and preventing metabolic inhibition (Jack et al. 2001; Tsuchiya et al. 2016). Additionally, genes related to the purine metabolism pathway were significantly downregulated in the biofilm state compared to the planktonic state. Purine metabolism is crucial for nucleotide synthesis, which drives bacterial proliferation (Liu et al. 2024). This downregulation reflected the reduced need for cell division and growth in biofilm‐associated bacteria, which exhibited slower proliferation rates and lower nucleotide requirements. The PTS, a key carbohydrate transport and phosphorylation system in bacteria, can facilitate carbohydrate uptake and phosphorylation for metabolic processing (Deutscher et al. 2006). This adaptation allowed bacteria to efficiently utilize limited carbohydrate resources in the biofilm, ensuring sufficient carbon and energy for stability and persistence. Moreover, by upregulating a thioredoxin‐related gene in the NOD‐like receptor signaling pathway, biofilm cells may mitigate oxidative stress within the biofilm and preserve dynamic equilibrium with the host immune system (Zeller and Klug 2006).

**TABLE 1:** Differentially expressed genes in biofilm formation of Li01 related KEGG pathways.

Term	Gene ID	Log_2_Fc	Type	Function
ABC transporters	Li01_GM000158	−1.189	Down	Methionine ABC transporter ATP‐binding protein
	Li01_GM000159	−1.601	Down	ABC transporter permease
	Li01_GM000160	−1.341	Down	MetQ/NlpA family ABC transporter substrate‐binding protein
	Li01_GM001445	−2.070	Down	Amino acid ABC transporter permease
	Li01_GM001446	−1.669	Down	Amino acid ABC transporter permease
	Li01_GM001447	−1.412	Down	Transporter substrate‐binding domain‐containing protein
	Li01_GM001448	−1.167	Down	Glutamine transport ATP‐binding protein
	Li01_GM001699	−1.128	Down	ABC transporter ATP‐binding protein
	Li01_GM001700	−1.109	Down	ABC transporter ATP‐binding protein
	Li01_GM001701	−1.088	Down	ABC transporter permease
	Li01_GM001702	−1.103	Down	Peptide ABC transporter permease
	Li01_GM001703	−1.007	Down	Peptide ABC transporter substrate‐binding protein
	Li01_GM001724	−1.204	Down	Oligopeptide ABC superfamily ATP binding cassette transporter substrate binding protein
One carbon pool by folate	Li01_GM000628	−1.231	Down	5‐formyltetrahydrofolate cyclo‐ligase
	Li01_GM000750	−5.199	Down	Bifunctional phosphoribosylaminoimidazolecarboxamide formyltransferase/IMP cyclohydrolase
	Li01_GM000749	−4.955	Down	Phosphoribosylglycinamide formyltransferase
	Li01_GM000852	−1.083	Down	Formate–tetrahydrofolate ligase
Quorum sensing	Li01_GM000353	1.205	Ups	DMT family transporter
	Li01_GM001702	−1.103	Down	Peptide ABC transporter permease
	Li01_GM001700	−1.109	Down	ABC transporter ATP‐binding protein
	Li01_GM001703	−1.007	Down	Peptide ABC transporter substrate‐binding protein
	Li01_GM001701	−1.088	Down	ABC transporter permease
	Li01_GM001699	−1.128	Down	ABC transporter ATP‐binding protein
	Li01_GM001724	−1.203	Down	Oligopeptide ABC superfamily ATP binding cassette transporter substrate binding protein
Purine metabolism	Li01_GM001316	−1.218	Down	Xanthine phosphoribosyltransferase
	Li01_GM000751	−5.357	Down	Phosphoribosylamine–glycine ligase
	Li01_GM000750	−5.198	Down	Bifunctional phosphoribosylaminoimidazolecarboxamide formyltransferase/IMP cyclohydrolase
	Li01_GM000748	−4.846	Down	Phosphoribosylformylglycinamidine cyclo‐ligase

	Li01_GM000749	−4.955	Down	Phosphoribosylglycinamide formyltransferase
	Li01_GM000747	−4.283	Down	Amidophosphoribosyltransferase
	Li01_GM000746	−3.846	Down	Phosphoribosylformylglycinamidine synthase subunit PurL
	Li01_GM000743	−2.964	Down	Phosphoribosylaminoimidazolesuccinocarboxamide synthase
Li01_GM000745	−3.544	Down	Phosphoribosylformylglycinamidine synthase subunit PurQ
Arginine biosynthesis	Li01_GM000326	1.579	Ups	Argininosuccinate lyase
	Li01_GM000327	1.081	Ups	Argininosuccinate synthase
Phosphotransferase system (PTS)	Li01_GM000106	1.198	Ups	PTS mannose/fructose/sorbose transporter subunit IIC
	Li01_GM000107	1.347	Ups	PTS mannose/fructose/sorbose transporter family subunit IID
	Li01_GM000802	1.136	Ups	PTS galactitol transporter subunit IIC
	Li01_GM001978	1.714	Ups	PTS glucitol/sorbitol transporter subunit IIA
	Li01_GM001979	1.537	Ups	PTS glucitol/sorbitol transporter subunit IIB
	Li01_GM001980	1.374	Ups	PTS glucitol/sorbitol transporter subunit IIC
RNA polymerase	Li01_GM000218	−1.428	Down	DNA‐directed RNA polymerase subunit beta
	Li01_GM000217	−1.260	Down	DNA‐directed RNA polymerase subunit beta
NOD‐like receptor signaling pathway	Li01_GM001123	1.070	Ups	Thioredoxin

*Note:* Differentially expressed genes (DEGs) were identified using the criteria: |Log_2_Fold Change| ≥ 1 and q‐value ≤ 0.05. DEGs with Log_2_Fold Change ≥ 1 were considered up‐regulated, and those with Log_2_Fold Change ≤ − 1 were considered down‐regulated in the biofilm state compared to the planktonic state.

Abbreviations: ABC transporters, ATP‐binding cassette transporter; Log_2_Fc, Log_2_Fold Change.

In summary, in the biofilm state, Li01 reduced its reliance on external resources and ensured long‐term survival in harsh gastrointestinal conditions through metabolic reprogramming, transport optimization, and efficient carbohydrate utilization.

## 4 Conclusions

This study provided a comprehensive analysis of the physiological characterization between planktonic and biofilm states of *Ligilactobacillus salivarius* Li01, *Bifidobacterium longum* , and *Bifidobacterium pseudocatenulatum* . The biofilm formation enhanced the survival of all strains under simulated gastrointestinal conditions, providing greater protection against gastric acidity and bile salts compared to their planktonic counterparts. Furthermore, biofilm formation influenced strain‐specific traits, including intestinal adhesion and surface properties. *L. salivarius* Li01 and *B. longum* showed enhanced adhesion, whereas *B. pseudocatenulatum* exhibited reduced adhesion due to lower hydrophobicity in the biofilm state. Therefore, the biofilm state does not universally enhance probiotic adhesion due to strain‐specific traits. Transcriptomic analysis of *L. salivarius* Li01 revealed distinct gene expression changes between the biofilm and planktonic states, particularly in pathways related to nutrient transport, metabolic adaptation, and quorum sensing. These changes reduced metabolic activity and optimized nutrient utilization, thereby enhancing stress resistance.

Overall, these findings suggest that the biofilm state of probiotics may be more effective in promoting gut colonization and enhancing resistance to gastrointestinal fluids compared to the planktonic state. Biofilm‐forming probiotics offer greater advantages for gastrointestinal delivery compared to their planktonic states, with potential applications in both treating and preventing gastrointestinal diseases. However, the study investigated the biofilm characteristics of only three probiotic strains, which may not represent the full variety of probiotic species. Future research should involve a broader range of probiotic strains to more comprehensively explore the benefits of biofilm formation.

## Author Contributions

**Wang Gao:** data curation (equal), formal analysis (equal), investigation (equal), methodology (equal), validation (equal), visualization (equal), writing – original draft (equal), writing – review and editing (equal). **Huijuan Jing:** data curation (equal), formal analysis (equal), investigation (equal), methodology (equal), validation (equal). **Bo Qiu:** data curation (supporting), formal analysis (supporting), investigation (supporting). **Shuobo Zhang:** formal analysis (supporting), investigation (supporting), visualization (supporting). **Jingyi Zhang:** formal analysis (supporting), investigation (supporting), visualization (supporting). **Lvwan Xu:** methodology (supporting). **Furong Ba:** methodology (supporting). **Siyuan Xie:** data curation (supporting). **Xiao‐Man Liu:** supervision (supporting). **Lanjuan Li:** conceptualization (equal), funding acquisition (equal), resources (supporting), supervision (supporting). **Mingfei Yao:** conceptualization (lead), funding acquisition (equal), resources (lead), supervision (lead).

## Conflicts of Interest

The authors declare no conflicts of interest.

## Data Availability

The data that supports the findings of this study are available from the corresponding author upon reasonable request.
